# Outer Membrane Vesicle Production by *Escherichia coli* Enhances Its Defense against Phage Infection

**DOI:** 10.3390/microorganisms12091836

**Published:** 2024-09-05

**Authors:** Guanhua Xuan, Di Lu, Hong Lin, Yinfeng Wang, Jingxue Wang

**Affiliations:** State Key Laboratory of Marine Food Processing & Safety Control, College of Food Science and Engineering, Ocean University of China, Qingdao 266400, China; xuanguanhua@ouc.edu.cn (G.X.);

**Keywords:** outer membrane vesicles, *E. coli*, phage, adsorption, resistance

## Abstract

Several studies have investigated the multifunctional characteristics of outer membrane vesicles (OMVs), but research on their role in mediating phage–bacteria interactions is limited. Employing *Escherichia coli* as a model, we engineered a mutant strain overproducing OMVs for protective experiments against phage infections. The addition of exogenous OMVs proved highly effective in safeguarding the bacterial host against various phages, mitigating predatory threats. Screening for phage-resistant strains and adsorption experiments revealed that inhibiting phage adsorption is a crucial pathway through which OMVs protect against phage predation. Although OMVs conferred tolerance to the phage-sensitive strains (those easily infected by phages), they could not restore the phage-resistant strains (those that effectively resist phage infection) to a sensitive phenotype. This study provides valuable insights for the future development of novel biotechnological approaches aimed at utilizing OMVs to protect fermentative strains and reduce the risk of phage contamination.

## 1. Introduction

Outer membrane vesicles (OMVs) are nano-sized spherical structures that are naturally released during the growth process of bacteria. Their diameters typically range from 20 to 300 nm, and their specific sizes can be influenced by various factors, including the type of bacteria, environmental conditions, and the mechanism of formation. These small vesicles carry a diverse array of biomolecules, including lipids, proteins, nucleic acids, and other cellular components [1,2,3]. Gram-negative bacteria have the inherent ability to produce OMVs under natural conditions, and the production of OMVs increases under conditions such as antimicrobial stress, nutrient deprivation, or bacterial cell lysis [4,5,6].

In recent years, substantial advancements have been achieved in comprehending the multifaceted functionalities of OMVs within microbial systems. Within microbial consortia, the intrinsic capacities of OMVs for encapsulation and delivery assume pivotal roles in bacterial intercellular communication [7]. Notably, OMVs exhibit the capability to sequester quorum sensing (QS) signaling molecules, thereby facilitating communication amidst bacterial communities and orchestrating the regulation of fundamental physiological processes, including, but not limited to, virulence and biofilm formation [8,9]. Furthermore, OMVs have been identified as instrumental in shielding bacterial cells from antibiotic-induced stress, especially when facing challenges with membrane-interfering peptides at certain concentrations [10,11]. The pivotal involvement of OMVs extends to their indispensable contributions to bacterial adaptation and resilience against external stressors, thereby assisting bacteria in mitigating environmental challenges and preserving their ecological niche [12,13].

Bacteriophages, specialized viruses targeting bacteria, pose a substantial threat to the structural integrity and equilibrium of microbial communities [14]. Numerous studies have shed light on the indispensable role of OMVs in safeguarding bacteria from phage assaults. For instance, OMVs released by *Vibrio cholerae* have demonstrated an ability to effectively counteract the intrusion of virulent phages ICP1, ICP2, and ICP3 through a dose-dependent and receptor-dependent baiting mechanism [15]. Analogous protective functions of OMVs have been elucidated in *Salmonella*, where it is theorized that OMVs may facilitate the transfer of phage DNA, thereby reducing the injection of phage particles into bacterial cells and fulfilling a defensive function [16]. OMVs derived from *Vibrio coralliilyticus* DSM 19607 demonstrated the inhibition of phage SBM1 infection in the *V. coralliilyticus* host, with impaired efficacy observed under elevated temperature conditions [17]. Despite isolated instances suggesting the potential of OMVs in defending against phages, the nature and functionalities of OMVs produced by different bacterial species vary considerably [18,19]. Moreover, further exploration of the intricate mechanisms underlying OMV-mediated resistance to phage infection remains relatively constrained.

In this study, we focused on elucidating the interactions between *Escherichia coli* and phages mediated by OMVs. Although an early and singular study reported rapid, irreversible binding between OMVs and T4 phages, facilitating *E. coli* defense against phage invasion [10], our study addresses previous limitations of single-phage testing models and methodological uniformity. By establishing multiple-phage infection models and employing diverse analytical approaches (such as adsorption efficiency assays and screening for OMV-overexpressing strains with acquired resistance to phages), this research delivers more robust conclusions. It deepens our understanding of bacterial defense mechanisms and provides a solid theoretical foundation for the future development of novel phage-resistant agents using OMVs, safeguarding fermentative strains from phage contamination.

## 2. Materials and Methods

### 2.1. Strains, Plasmids, and Growth Conditions

All bacterial strains and plasmids are listed in Supplementary Table S1, and all primers are given in Supplementary Table S2. Gene-deletion mutants of *E. coli* MG1655 were constructed by using a reported one-step deletion method [20] with long homology arms to enhance the success rate. All *E. coli* strains were cultured in LB medium at 37 °C or 25 °C with shaking (200 rpm). Ampicillin (100 µg/mL) or kanamycin (50 µg/mL) were added as needed. Phages specific to *E. coli* were isolated from sewage samples collected in Qingdao, China.

### 2.2. OMV Separation, Quantitation, and Characterization

OMVs were prepared using a previously reported method with certain modifications [21]. In brief, the *E. coli* MG1655∆*nlpI*∆*tolA* strain was cultured overnight at 37 °C with shaking (200 rpm) in LB broth. On the subsequent day, the overnight culture was transferred to fresh LB medium at a 1% ratio and cultivated at 37 °C with agitation at 200 rpm until reaching an OD_600nm_ of approximately 0.7. Following this, a centrifugation step (10,000× *g*, 20 min, 4 °C) and subsequent membrane filtration (0.45 μm pore size) were employed to eliminate bacterial cells and debris from the culture supernatant. The resulting filtrate underwent ultracentrifugation (110,000× *g*, 2 h, 4 °C), and the resulting pellets were washed with pre-chilled Phosphate-Buffered Saline (PBS) buffer. The above ultracentrifugation steps were repeated once to achieve the OMVs.

The quantification of the OMV yield was conducted through the measurement of their protein content per colony-forming unit (CFU). The total protein concentration was determined by a Bicinchoninic Acid Assay (BCA) Protein Assay Kit (Solarbio Science & Technology Co., Ltd., Beijing, China) according to the manufacturer’s instructions. The size analysis of the enriched OMVs was conducted using a nanoparticle size analyzer (nano zs90), and the particle size distribution of the OMVs was examined using TEM and ImageJ (version 1.53q).

### 2.3. Transmission Electron Microscope (TEM) Analysis

The prepared OMV samples were applied to carbon-coated copper grids and allowed to adsorb for 5 min, and excess liquid was eliminated by blotting with filter paper. For the observation of phage adsorption to the OMVs, samples of 500 μL of OMVs (0.3 mg/mL) were mixed with an equal volume of phage solution (10^5^ PFU/mL). The mixture was thoroughly blended and then incubated at 37 °C for 15 min. Samples were loaded onto a carbon-coated copper grid for 5 min, followed by negative staining using 2% phosphotungstic acid (pH 6.8) for 2 min. Following a 5 min air-drying period at room temperature, the samples were observed and examined using a JEM-2000EX (JEOL, Tokyo, Japan) transmission electron microscope operating at 100 kV.

### 2.4. Phage Resistance Assay

Spot assay: For the preparation of double-layer agar plates, 100 µL overnight cultures of *E. coli* strains (2.4 × 10^8^ CFU/mL) were individually added to 5 mL of semi-solid LB medium. To investigate whether the exogenously added OMVs could protect the bacteria against phage infection, a mixture of 100 μL of phage IME339 solution (10^9^ PFU/mL) and 900 μL of the prepared OMVs (0.25 mg/mL) was incubated for 15 min. A control was set up by adding an equal volume of PBS buffer without OMVs. Subsequently, 4 μL samples of the phage suspension with serial dilutions (10^6^, 10^5^, 10^4^, and 10^3^ PFU/mL) were spotted on the double-layer plates. After air-drying, the inverted plates were incubated at 37 °C for 10 h without agitation. The lytic ability of the phages against different hosts was determined by observing and recording the transparency of the phage plaques. The relative efficiency of plating (EOP) was calculated by dividing the average PFU count on the target bacteria by the average PFU count on the control bacteria.

Bacterial growth reduction assay: An overnight culture of the *E. coli* strains was diluted 1% into fresh LB medium and incubated at 37 °C with agitation at 200 rpm until the bacterial concentration reached approximately 10^8^ CFU/mL. Subsequently, phage IME339 was introduced into the culture at a multiplicity of infection (MOI) of 0.1. A 100 µL aliquot of the resulting mixture was promptly transferred to a sterile 96-well plate containing 100 µL of either PBS (control group) or OMVs (experimental group). The absorbance values at OD_600nm_ were determined using a microplate reader, and the plate was further incubated at 37 °C with continuous shaking at 200 rpm. The OD_600nm_ values were measured and recorded at regular intervals. The same approach was used to explore the OMVs’ protective capacity against various phage infections in MG1655. Notably, IME339 was introduced at MOI = 0.1, IME347 and PZJ0206 at MOI = 0.5, and IME281 at MOI = 0.05 to assess the bacterial growth reduction.

### 2.5. Preparation of Strain with Resistance to Phages

Overnight-cultured MG1655∆*nlpI*∆*tolA* strains were transferred at a 1% ratio into fresh LB medium supplemented with a high titer of phage IME339 (MOI = 10). The co-culture was incubated at 37 °C and at 200 rpm for 24 h. Subsequently, 2 mL of the culture was centrifuged at 9660× *g* for 5 min, and the supernatant was discarded. The pellet was resuspended in sterile PBS, and 2 mL of the resuspended material was added to a new 50 mL LB medium with additional high-titer phage (MOI = 10) for another 24 h co-culture. This process was repeated with centrifugation, resuspension, and streaking the resuspended material onto agar plates. Then, the single-colony resistant strain was selected and purified by streaking. The acquired resistant strain, named MG1655∆*nlpI*∆*tolA*-R, was further verified by classic spot tests, as described.

### 2.6. Adsorption Rate Assay

Overnight cultures of *E. coli* strains were diluted 1:100 and cultured in fresh LB medium overnight. To promote phage adsorption, 0.5 mL of a phage solution (10^5^ PFU/mL) was mixed with 0.5 mL of the cell suspension (10^8^ CFU/mL) and incubated at 37 °C for 15 min. As a control, PBS buffer mixed with phage IME339 without bacteria was used. To investigate the direct adsorption effect of phages on OMVs, samples of 0.5 mL of the OMVs (0.25 mg/mL) were mixed with 0.5 mL of phage dilution (10^5^ PFU/mL) and co-incubated for 15 min. Following incubation, the cultures were centrifuged at 9660× *g* for 3 min, and the titer of free phage in the supernatant was determined using the double-layer agar method. The phage adsorption rate was calculated as follows: adsorption rate (%) = [(initial phage titer—phage titer in the supernatant)/(initial phage titer)] × 100.

### 2.7. Statistical Analysis

Unless explicitly mentioned, bar charts depict the mean ± SD derived from a minimum of three independent biological experiments. Statistical analysis and graph plotting employed the GraphPad Prism 8.0 and Origin 9.80 software, with group differences evaluated using an unpaired *t*-test.

## 3. Results

### 3.1. Separation and Characterization Analysis of E. coli OMVs

**Figure 1:**
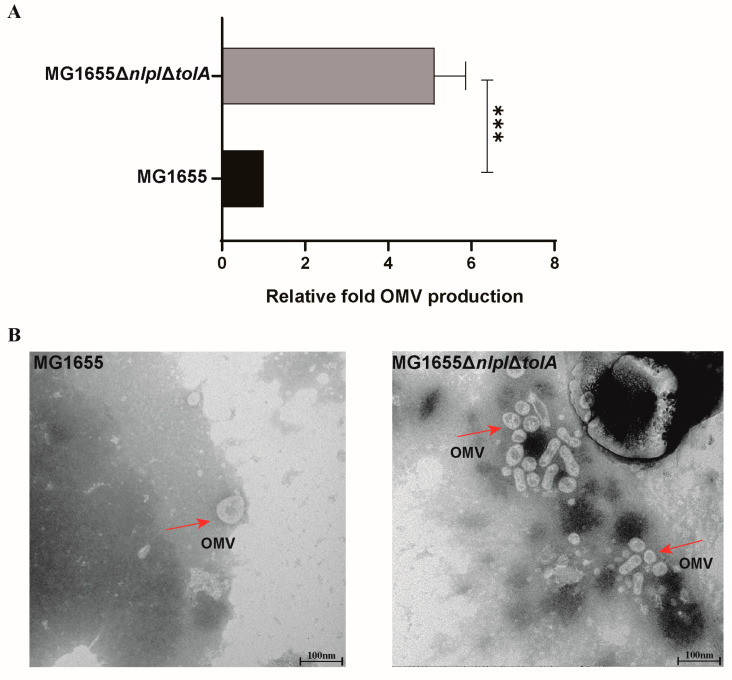
The *E. coli* MG1655∆*nlpI*∆*tolA* strain overproduces OMVs. (**A**) Comparison of the yield of OMVs between MG1655∆*nlpI*∆*tolA* and wild-type MG1655. Mean values ± standard deviation were calculated from three independent experiments. A *t*-test was performed (***, 0.0001 < *p* < 0.001). (**B**) TEM images of OMVs from MG1655∆*nlpI*∆*tolA* compared to wild-type MG1655 (left image: MG1655; right image: MG1655∆*nlpI*∆*tolA*). The red arrow indicates the formed OMVs.

The gene product encoded by *nlpI*, NlpI, is an outer membrane (OM)-anchored lipoprotein involved in cell division and participates in regulating peptidoglycan (PG) synthesis metabolism [22]. The *tolA* gene encodes TolA, an inner membrane protein that is an integral component of the bacterial envelope Tol-Pal system. Mutations in Tol-Pal disrupt the OM permeability barrier, leading to the release of periplasmic proteins and the formation of OMVs [23]. The correlation between the *nlpI* and *tolA* genes and the quantity of OMVs has been widely investigated [24,25]. By generating endogenous *nlpI* and *tolA* gene-deletion strains, it was discovered that there was a substantial increase in OMV production (Figure 1A). TEM analysis comparing the wild-type strain MG1655 with the mutant strain MG1655∆*nlpI*∆*tolA* revealed that despite the increased OMV yield in the mutant strain, there was minimal change in their morphological size (Figure 1B).

**Figure 2:**
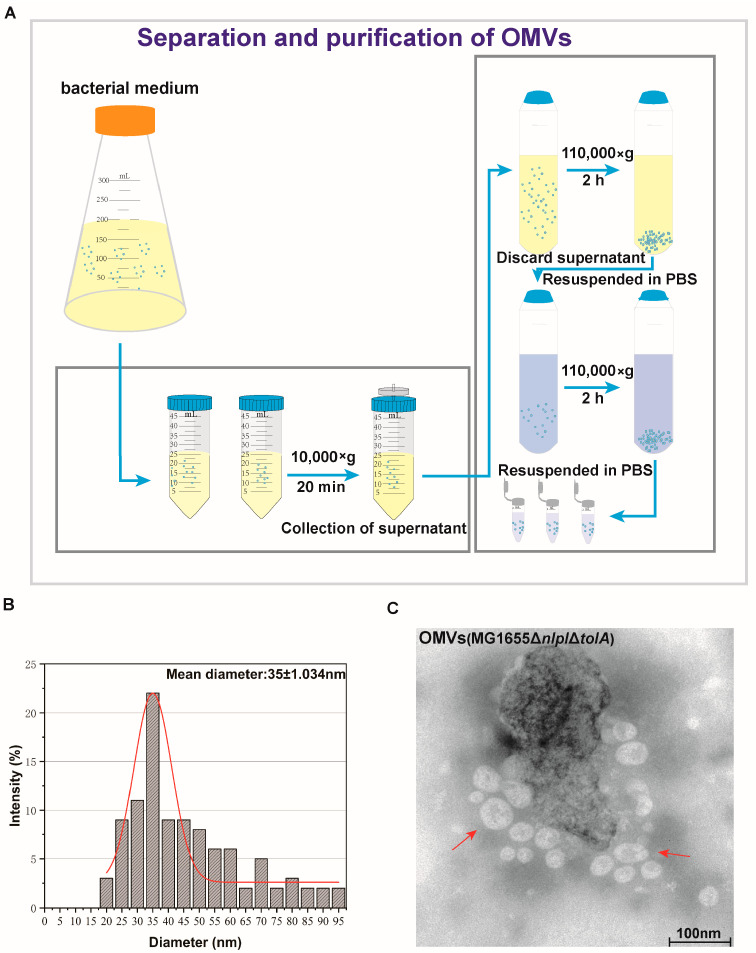
Preparation and characterization of OMVs. (**A**) Preparation process of OMVs from *E. coli*. (**B**) Size distribution of *E. coli* OMVs. (**C**) TEM images of *E. coli* OMVs. The red arrow indicates the formed OMVs.

The investigation utilized centrifugation as a method for the separation and preparation of OMVs from *E. coli*, as depicted in Figure 2A. Analysis of the OMVs’ particle size revealed a size range of 20–100 nm, with a peak at 35 nm (Figure 2B). Further examination through TEM showcased the morphology and size of the isolated OMVs, displaying spherical particles with a diameter of approximately 45 nm (Figure 2C). These findings were similar to the results obtained from the nanoparticle size analyzer (Figure 2B) and affirm the successful isolation of spherical double-layered membrane OMVs from *E. coli* under in vitro cultivation conditions.

**Figure 3:**
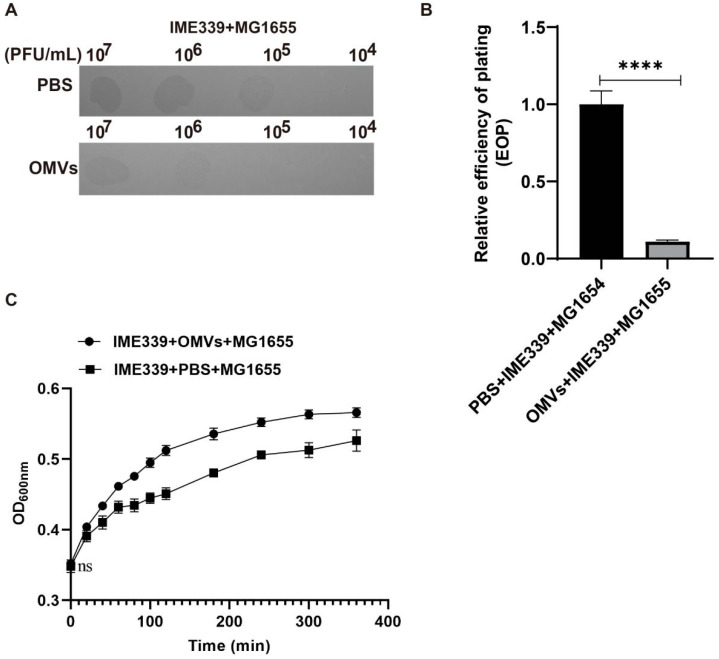
Host survival under phage predation pressure was markedly improved by the external introduction of prepared OMVs. (**A**) Assessing the influence of exogenous OMV addition on phage plaque formation on *E. coli.* Series dilutions of 4 μL of phage IME339 were spotted on MG1655 for plaque-forming ability determination. (**B**) Relative EOP of phage IME339 on *E. coli* MG1655 before and after addition of OMVs. Mean values ± standard deviation were calculated from three independent experiments. A *t*-test was performed (****, *p* < 0.0001). (**C**) Growth curves were determined at different time points after infecting *E. coli* MG1655 strains with phage IME339 under the conditions of exogenously added OMVs. The optical density (OD) at 600 nm was measured using a SynergyH1 microplate reader in a 96-well plate.

The prepared OMVs were employed to treat *E. coli*. It was observed that the OMV-treated *E. coli* exhibited heightened resistance against phage infection as compared to the control group treated with PBS (Figure 3A). Relative EOP calculations demonstrated a significantly lower EOP for phage IME339 on the OMV-treated *E. coli* (Figure 3B). The co-growth curves further confirmed that the exogenously added OMVs markedly enhanced *E. coli’s* resistance against phage invasion (Figure 3C).

**Figure 4:**
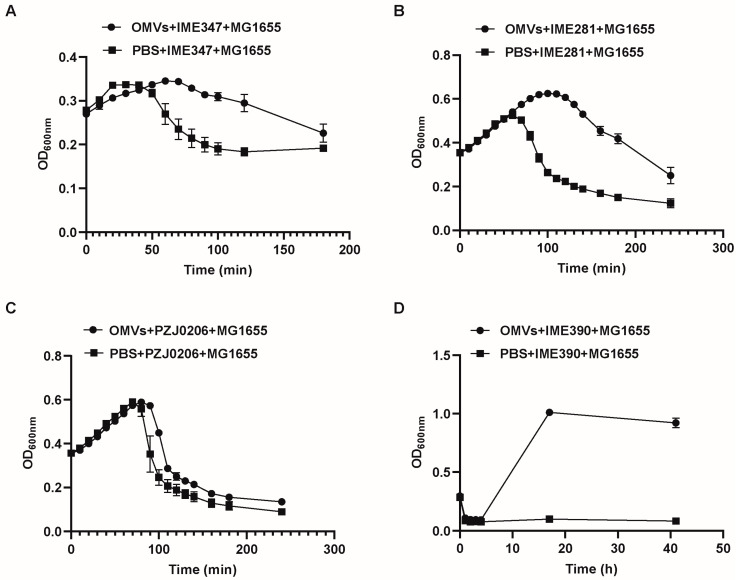
Examining the role of exogenously added OMVs in the defense of host *E. coli* MG1655 against infections by different phages. Growth curves were determined at different time points after infecting *E. coli* MG1655 strains with (**A**) phage IME347, (**B**) phage IME281, (**C**) phage PZJ0206, and (**D**) phage IME390 under the conditions of exogenously added OMVs.

Beyond the phage IME339, the OMVs could confer protection to the host against various phage infections (IME281, IME390, IME347, and PZJ0206). According to reports, IME339 is most closely related to the genus *T4virus*, family *Myoviridae*; IME347 is most closely related to the genus *T1virus*, family *Siphoviridae*; PZJ0206 is most closely related to the genus *Berlinvirus*, family *Autographiviridae*; IME390 is most closely related to the genus *Teseptimavirus*, family *Autographiviridae*; and IME281 is most closely related to the genus *Js98virus*, family *Myoviridae* [26,27,28]. Therefore, the OMVs exhibited relatively broad-spectrum characteristics in their phage-resistance capabilities (Figure 4).

### 3.2. OMVs Act Primarily by Hindering Phage Adsorption to Host Cells

**Figure 5:**
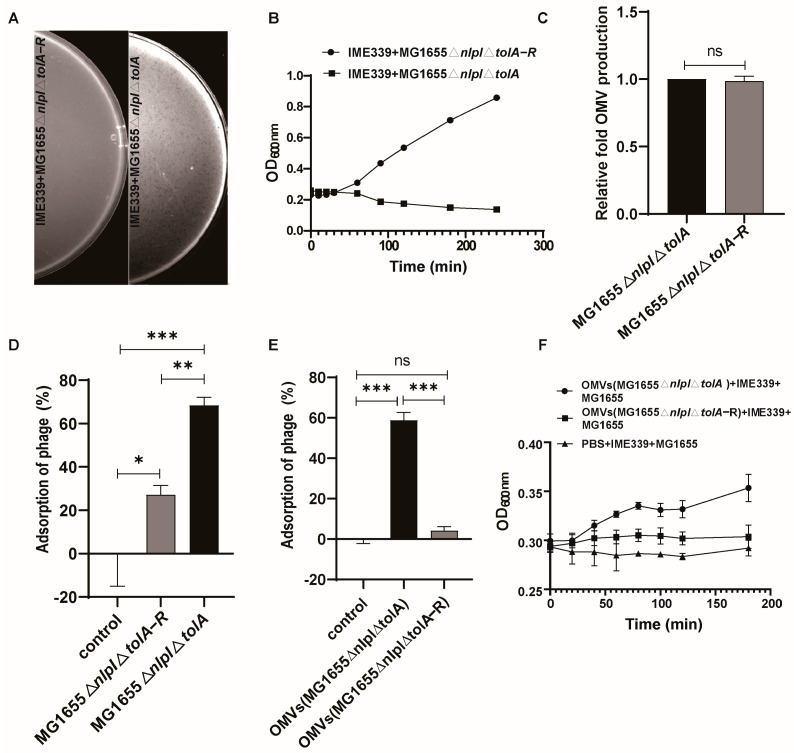
OMVs can provide multiple binding targets for phages, thereby disrupting phage invasion of the host. (**A**) Plaque assay characterizing the phage-resistant strain MG1655∆*nlpI*∆*tolA*−R. (**B**) Co-culture growth curves testing the phage-resistant strain MG1655∆*nlpI*∆*tolA*−R. (**C**) Analysis of the OMV production capacity in strains MG1655∆*nlpI*∆*tolA* and MG1655∆*nlpI*∆*tolA*−R. (**D**) Adsorption experiment of phage IME339 on MG1655∆*nlpI*∆*tolA* and MG1655∆*nlpI*∆*tolA*−R. (**E**) Adsorption experiment of phage IME339 on the OMVs secreted by strains MG1655∆*nlpI*∆*tolA* and MG1655∆*nlpI*∆*tolA*−R. (**F**) Co-culture growth curve analysis determining the impact of OMVs from MG1655∆*nlpI*∆*tolA* and MG1655∆*nlpI*∆*tolA*−R on *E. coli*’s resistance to phages. Mean values ± standard deviation were calculated from three independent experiments. A *t*-test was performed (“ns”, no significance; *, 0.01 < *p* < 0.05; **, 0.001 < *p* < 0.01; ***, 0.0001 < *p* < 0.001).

To elucidate the protective mechanism conferred by OMVs, we directed the evolution of MG1655∆*nlpI*∆*tolA*, screening and obtaining a strain with enhanced resistance to phage invasion, denoted as MG1655∆*nlpI*∆*tolA*-R. Further validation through a spot assay (Figure 5A) and co-culture growth curve assays (Figure 5B) confirmed the complete resistance of MG1655∆*nlpI*∆*tolA*-R to phage IME339. Although the production of OMVs in the resistant strain showed no significant changes (Figure 5C), the adsorption efficiency of phages to the resistant strain was markedly reduced compared to MG1655∆*nlpI*∆*tolA* (Figure 5D). Subsequent preparation of OMVs from MG1655∆*nlpI*∆*tolA* and MG1655∆*nlpI*∆*tolA*-R, followed by assessing the adsorption efficiency of phages to these OMVs, revealed that phage IME339 effectively adhered to MG1655∆*nlpI*∆*tolA*, while the adsorption rate to OMVs from MG1655∆*nlpI*∆*tolA*-R was extremely low, resembling the control group without OMVs (Figure 5E). The co-culture growth curve results indicated that the externally added OMVs from MG1655∆*nlpI*∆*tolA*-R contributed minimally to the protective effect on the host compared to the OMVs from MG1655∆*nlpI*∆*tolA* (Figure 5F). Similar to the host, OMVs possess crucial target sites for phage recognition, leading us to hypothesize that preventing effective phage adsorption is a pivotal mechanism by which OMVs protect the host, inducing tolerance to phages.

**Figure 6:**
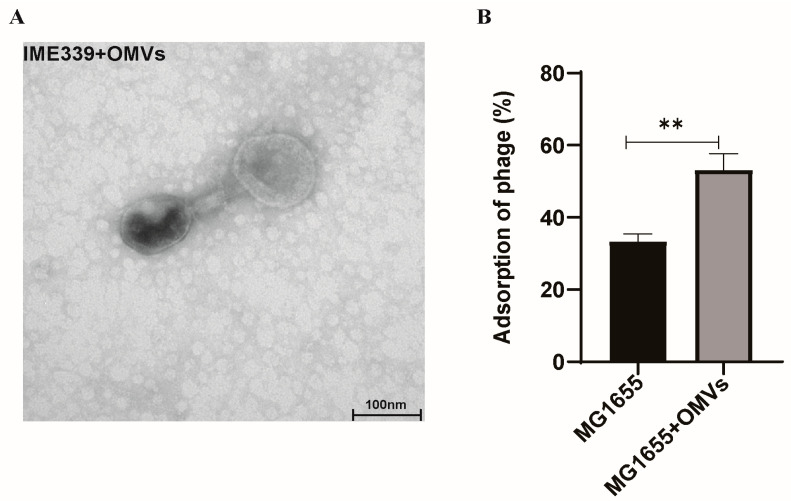
Phage adsorption assays. (**A**) TEM observation of phage IME339 adsorption on OMVs. (**B**) OMVs were introduced into the culture of *E. coli* MG1655, and changes in the adsorption efficiency of phage IME339 were assessed. Mean values ± standard deviation were calculated from three independent experiments. A *t*-test was performed (**, 0.001 < *p* < 0.01).

The study conducted using TEM demonstrated the ability of phage IME339 to successfully adsorb onto the OMV surface (Figure 6A). This emphasizes the importance of the OMV surface as a critical recognition target for phages. Furthermore, the OMV-treated group in the MG1655 culture exhibited a markedly elevated phage adsorption efficiency compared to the control group without OMV treatment (Figure 6B). These findings suggest that OMVs represent essential targets for phage adsorption, playing a significant role in phage neutralization.

### 3.3. OMVs Affiliated with Resistant Bacterial Cells Exhibit Insensitivity to Phage Infection

**Figure 7:**
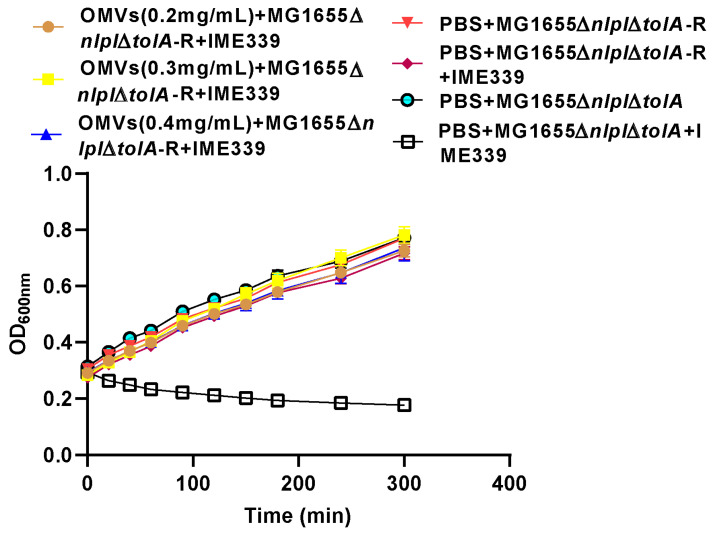
OMVs do not facilitate the effective restoration of phage-resistant strains to a sensitive phenotype. Co-culture growth curve experiments were conducted to analyze the impact of varying OMV concentrations on the conversion between phage-resistant and phage-sensitive phenotypes. The OD_600nm_ was measured using a SynergyH1 microplate reader in a 96-well plate.

The results indicate that the excessive production of vesicles in strain MG1655∆*nlpI*∆*tolA* and its resistant variant MG1655∆*nlpI*∆*tolA*-R exhibited minimal growth disparities. The incorporation of OMVs from the non-resistant strain MG1655∆*nlpI*∆*tolA* into the phage-resistant strain MG1655∆*nlpI*∆*tolA*-R did not result in the restoration or partial restoration of sensitivity to phages, even at high OMV concentrations (0.4 mg/mL), as evidenced by the co-growth curve analysis (Figure 7). This indicates that OMVs cannot effectively revert a phage-resistant strain to a phage-sensitive phenotype (one-way functionality).

## 4. Discussion

This study provides substantial evidence confirming the ability of OMVs to offer effective host protection against phage invasion in *E. coli*. The inhibition of phage adsorption to the host bacteria was identified as a crucial pathway through which OMVs exert their protective role (Figure 5 and Figure 6). While the exogenous addition of OMVs effectively protected the host against the phage, it failed to restore the phage-sensitive phenotype in resistant strains (Figure 7). The excessive production of OMVs by bacteria is often closely related to environmental adaptation, and our research also supports this argument that OMVs have important significance in microbial ecosystems, and our findings contribute to a deeper understanding of OMV-mediated phage–bacteria interactions.

In reality, numerous studies have explored the relationship between OMVs and phages, but many have focused on identifying and proving that phage infection is a significant inducer of OMV production [29,30]. Few have reported the crucial role of OMVs in protecting the host against phage attacks. Derived from bacteria, OMVs share a structural similarity with the bacterial membrane architecture [31,32,33], determining their crucial role as recognition and adsorption targets for phages. Our data reveal that OMVs from receptor-mutant phage-tolerant strains fail to provide effective phage adsorption targets (Figure 5F). Furthermore, the results indicate that phages can directly adsorb to the surface of OMVs from phage-sensitive strains (Figure 6). This highlights the essential pathway through which OMVs act, providing ineffective adsorption targets that efficiently isolate phage invasion, thus offering protection to the host. Given the widespread secretion of OMVs by bacteria in nature, especially under adverse environmental conditions [13,34], this suggests a significant strategy for bacteria to adapt to predation pressure from phages and survive.

External addition of *E. coli* OMVs can provide protection for the host against various phage infections (Figure 4), and this protection extends to future preparations and combinations of OMVs from different bacterial strains, broadening the application range and capability of OMVs in phage defense. Despite the remarkable effectiveness of OMVs in phage resistance, they could not efficiently restore phage sensitivity in the mutant strain MG1655∆*nlpI*∆*tolA*-R (Figure 7).

In summary, our findings provide compelling evidence of the anti-phage capabilities of OMVs, enhancing our understanding of their diverse functions. However, further research is warranted to explore the specific roles of OMVs within complex microbial communities closely simulating real ecological conditions. This investigation will be instrumental in providing essential theoretical support for future applications targeting OMVs.

## 5. Conclusions

Traditional research on OMVs has focused on factors inducing their production, their functions, and their adaptation to extreme environments, with limited understanding of their role in bacteria–phage interactions. In our study, using *E. coli* OMVs as a starting point, we discovered that OMVs can serve as “bait” for phages, acting as a protective barrier for the host, thereby reducing phage invasion. In natural environments, phages pose a significant threat to bacteria, often leading to explosive overproduction of OMVs upon phage infection. Despite this, the generated OMVs can still effectively bind to phages, providing a defense mechanism for bacterial populations. This research elucidates the importance of OMVs in the complex interactions between bacteria and phages, expanding our understanding of OMV functions. This has significant implications for exploring the intricate interplay between bacteria and phages, developing novel antibacterial strategies, and gaining deeper insights into the stability of microbial ecosystems.

## Data Availability

All data presented in this work are available upon request. The *E. coli* phages IME339 (accession number MH051915), IME281 (accession number MH051913), IME390 (accession number MH779619), IME347 (accession number MH051918), and PZJ0206 (accession number MT625440) have been sequenced and deposited in NCBI GenBank.

## Supplementary Materials

The following supporting information can be downloaded at: https://www.mdpi.com/article/10.3390/microorganisms12091836/s1, Table S1: Strains and plasmids used in this study; Table S2: Primers used in this study.
