# Supplementary material for: Outer Membrane Vesicle Production by Escherichia coli Enhances Its Defense against Phage Infection
Source: Microorganisms. 2024 Sep 5;12(9):1836. doi: 10.3390/microorganisms12091836 (PMC11433858; doi:10.3390/microorganisms12091836)
Supplement: Supplementary file 1 [file microorganisms-12-01836-s001.zip › microorganisms-3189501-supplementary.pdf]

Supplemental Information

Table S1 Strains and plasmids used in this study.

Strain	Characteristic	Source
E. coli DH5α	Cloning strain	Invitrogen
E. coli MG1655	wild type, used as chassis for protein express in physiological level.	Invitrogen
E. coli Δ*nlpI*	*nlpI* deletion of *E. coli* MG1655	This study
E. coli Δ*tolA*	*tolA* deletion of *E. coli* MG1655	This study
E. coli Δ*nlpI*Δ*tolA*	*nlpI* and *tolA* deletion of *E. coli* MG1655	This study
E. coli Δ*nlpI*Δ*tolA*-R	phage-resistant strain	This study
**Plasmid**	**Characteristic**	**Source**
pTK-Red	Spc^+, temperature-sensitive, used for gene knockout in *E. coli*	Invitrogen
pCP20	Amp^+, temperature-sensitive, used for gene knockout in *E. coli*	Invitrogen

Table S2 Primers used in this study.

Primers	Sequence (5'-3')	Usage
nlpI-wanner50-fr	CGGGAACAGGACGTTCATTCAACCGTGGTCTTCGGGAGTGGGAAATGAAGTGTAGGCTGGAGCTGCTTCG	PCR product was used for *nlpI* gene knockout in MG1655 strain
nlpI-wanner50-R	CAAAAAAGATTACGGGCTGATGTGTACGTCAGCTATTGCTGGTCCGATTCATGGGAATTAGCCATGGTCC	
nlpI-verify-F	CGACTGAGCAGTCTCAACCTGC	Verification of *nlpI* gene knockout
nlpI-verify-R	GCGGCCAGTCTACATAACTCATC	
tolA-wanner50-fr	TGAAAGAGAGCGGGTAACAGGCGAACAGTTTTTGGAAACCGAGAGTGTCATGTAGGCTGGAGCTGCTTCG	PCR product was used for *tolA* gene knockout in MG1655 strain
tolA--wanner50-R	GATGTTGACCGTCCGAACAGTCAACATCGCGATTACGGTTTGAAGTCCAAATGGGAATTAGCCATGGTCC	
tolA-verify-F	CAACCCGAAAACGGTCTTTCTGAT	Verification of *tolA* gene knockout
tolA-verify-R	TGGCCCACGATAATTTAGCAGAATG	
